# Serum Cytokines Predict the Severity of Coronary Artery Disease Without Acute Myocardial Infarction

**DOI:** 10.3389/fcvm.2022.896810

**Published:** 2022-05-16

**Authors:** Sheng Liu, Chenyang Wang, Jinzhu Guo, Yunxiao Yang, Mengling Huang, Li Li, Yu Wang, Yanwen Qin, Ming Zhang

**Affiliations:** ^1^Center for Coronary Heart Disease, Beijing Anzhen Hospital, Capital Medical University, Beijing, China; ^2^Department of Cardiology, Baotou Jiuyuan District Hospital, Baotou, China; ^3^Surgical Center of Structural Heart Disease, Beijing Anzhen Hospital, Capital Medical University, Beijing, China; ^4^Liver Research Center, Beijing Friendship Hospital, Capital Medical University, Beijing, China; ^5^Key Laboratory of Upper Airway Dysfunction-related Cardiovascular Diseases, Beijing Institute of Heart, Lung and Blood Vessel Disease, Beijing Anzhen Hospital, Capital Medical University, Beijing, China

**Keywords:** cytokines, interleukin-12 (IL-12), interleukin-17 (IL-17), coronary artery disease, high-density lipoprotein cholesterol (HDL-C)

## Abstract

**Introduction:**

Various cytokines were involved in the process of atherosclerosis, and their serum levels were correlated with coronary artery disease (CAD) to varying degrees. However, there were limited reports about the correlation between serum cytokines and the severity of coronary atherosclerotic lesion in patients with non-acute myocardial infarction (AMI). The purpose of this study was to investigate the relationship between serum cytokines and the severity of CAD, and identify the predictors of severe CAD in patients suspected to have CAD but AMI had been ruled out.

**Methods:**

A total of 502 patients who had suspected CAD and underwent coronary angiography were enrolled. The serum levels of IL-1β, IL-2, IL-4, IL-5, IL-6, IL-8, IL-10, IL-12p70, IL-17, TNF-α, IFN-α,and IFN-γ were determined by multiplexed particle-based flow cytometric assays technology. And the severity of CAD was evaluated by Gensini score (GS).

**Results:**

The serum levels of IL-4, IL-12p70, IL-17, and IFN-α were significantly lower in the severe CAD group (GS≥30) than those in the non-severe CAD group (GS < 30). And IL-12p70 and IL-17 were negatively correlated with the severity of CAD. Multivariate logistic regression analyses demonstrated that two serum cytokines (IL-12p70 and IL-17), one clinical protective factor (HDL-C), and two clinical risk factors (gender and diabetes) were the independent predictors of severe CAD. ROC curve analysis showed that multivariate mode combined these predictors had a good performance in predicting severe CAD.

**Conclusion:**

The combination of serum cytokines (IL-12p70 and IL-17) and clinical risk factors (HDL-C, gender, and diabetes) may help identify patients with more severe coronary artery lesions from those with suspected CAD but not AMI, and may contribute to guiding the risk stratification for patients with chest discomfort in health care facilities without sufficient medical resources (especially cardiac catheterization resources).

## Introduction

Coronary artery disease (CAD) is a chronic inflammatory disease characterized by atherosclerosis of the coronary arteries and a serious health threat worldwide. It had been proved that the severity of CAD was associated with cardiovascular death and all-cause mortality (1, 2). Therefore, identifying the predictors of severe CAD is helpful for the diagnosis and treatment of CAD. It is known that inflammation drives the development of atherosclerosis which is the main pathological feature of CAD (3), and as inflammatory markers, various cytokines were found to be extensively involved in the process of atherosclerosis (4, 5). It is reported that the elevated concentration of interleukin-1beta (IL-1β), Interleukin-2 (IL-2), interleukin-6 (IL-6), interleukin-8 (IL-8), interleukin-12p70 (IL-12p70), tumor necrosis factor-alpha (TNF-α), and interferon-gamma (IFN-γ) were associated with the increased risks of acute coronary syndrome (ACS) (6–9). Interleukin-5 (IL-5) and interleukin-10 (IL-10) were thought to be protective factors against atherosclerosis. Interferon- alpha (IFN-α) may promote the development of atherosclerosis potentially in individuals with systemic lupus erythematosus (SLE) (10). In addition, the roles played by some cytokines such as interleukins-4 (IL-4) and interleukin-17 (IL-17) in CAD is controversial, elevated levels of IL-4 and IL-17 had been reported in patients with ACS (11, 12), however, it had also been suggested that they may exert atheroprotective effects (13, 14).

Previous studies had tended to investigate the differences in serum cytokines levels between patients with and without CAD, and fewer studies had linked cytokines levels to the severity of coronary atherosclerotic plaque burden. Furthermore, most previous studies did not exclude patients with acute myocardial infarction (AMI), but included them in the analysis of patients with CAD, whereas in fact AMI would cause dramatic changes in cytokine levels (15–18), which may make the conclusions inaccurate. The purpose of this study was to investigate the relationship between serum cytokines (IL-1β, IL-2, IL-4, IL-5, IL-6, IL-8, IL-10, IL-12p70, IL-17, TNF-α, IFN-α, and IFN-γ) and the severity of CAD, and determine the predictors of severe CAD to identify patients with more severe coronary artery lesions from people suspected to have CAD but AMI had been ruled out.

## Materials and Methods

### Study Population

A total of 502 hospitalized patients of the cardiovascular department of Beijing Anzhen Hospital were successively enrolled in this study from March 12, 2021 to October 30, 2021. All patients were suspected of having CAD because of the symptoms of typical or atypical chest discomfort and underwent percutaneous coronary angiography (CAG). Patients were divided into CAD group and non-CAD group according to results of CAG. The diagnostic criteria for CAD was the presence of severe stenosis (>50%) in at least one major coronary artery segment by CAG analysis. Assessed by the Gensini score system, Patients with CAD were further divided into mild CAD group (GS < 30) and severe CAD group (GS≥30), and the non-severe CAD group included both the non-CAD group and the mild CAD group. Patients with acute myocardial infarction (AMI) were excluded, and the diagnosis of AMI was according to “Fourth Universal Definition of Myocardial Infarction” (19). Other exclusion criteria included valvular heart disease, myocardiopathy, severe heart failure, severe arrhythmia, active inflammatory or infectious disease, autoimmune disease, malignant tumor, severe hepatic and renal dysfunction, and taking anti-inflammatory medicine. This research has been authorized and registered by the Medical Ethics Ming Zhang, zhangming2279@hotmail.com Committee of Beijing Anzhen Hospital in accordance with the Declaration of Helsinki and all study patients have written informed consent.

The venous blood samples were collected from each patient after at least 12-h fasting in the morning before CAG. After centrifugation (2,500 g for 10 min) of the blood sample, the serum was collected and stored at −80°C for cytokine assay. All procedures were completed within 1 h.

### Demographic and Clinical Information

The clinical characteristics of each patient including gender, age, Body Mass Index (BMI), the history of hypertension, hyperlipidemia, diabetes, and smoking, and the usage of aspirin, statin, and angiotensin converting enzyme inhibitors (ACEI) or angiotensin receptor blocker (ARB) were recorded before CAG. The biochemical parameters including total triglycerides (TG), total cholesterol (TC), high-density lipoprotein cholesterol (HDL-C), low-density lipoprotein cholesterol (LDL-C), non-HDL-C, high-sensitive C-reactive protein (hsCRP), homocysteine (Hcy), urea, serum creatine (Scr), effect glomerular filtration rate (eGFR), uric acid (UA), fasting blood glucose (FBG) and high-sensitive troponin I (hsTnI) were analyzed in the Department of Biochemical Laboratory at Beijing Anzhen Hospital. These parameters were measured by a biochemical analyzer (Hitachi-7600, Tokyo, Japan) and chemiluminescence immunoanalyzer (Abbott-i2000SR, Illinois, USA) using blinded quality control specimens. The intra-assay and inter-assay coefficients of variation were < 5 and < 10%, respectively.

### Cytokine Assay

The concentrations of the serum cytokines (IL-1β, IL-2, IL-4, IL-5, IL-6, IL-8, IL-10, IL-12p70, IL-17, TNF-α, IFN-α, and IFN-γ) were determined by multiplexed particle-based flow cytometric assays technology on the BD FACSCanto II system (BD Biosciences, San Jose, CA, USA), using a 12-in-1 Human Cytokine Assay Kit (Ruisikeer Biotechnology, Laoshan, Qingdao, China), and the further analysis using FACSCanto Clinical Software (BD Biosciences, San Jose, CA, USA). All analyses were performed in accordance with the manufacturer's instructions. After generating the standard curves for each cytokine, the concentration of each cytokine in each well was converted from the mean fluorescence intensity by using the linear portion of the standard curves.

### Coronary Angiography and Gensini Score Calculation

All patients underwent CAG, and two independent experienced interventional cardiologists determined whether the patient had CAD and evaluated the severity of coronary artery stenosis by the GS system based on the images of CAG. GS was scored according to the position and severity of coronary artery stenosis, a score of 1, 2, 4, 8, 16, and 32 correspond to 25, 50, 75, 90, 99% obstruction, and complete occlusion (100 %) of coronary artery, respectively. And according to the functional significance of the area supplied by each segment, a multiplying factor was applied to each lesion score based upon its location in the coronary tree. Specifically, in the right dominant coronary system, 5 for the left main coronary artery, 2.5 for the proximal segment of left circumflex artery (LCX) or proximal segment of left anterior descending artery (LAD), 1.5 for the mid segment of LAD, 1 for the mid and distal segment of LCX, obtuse marginal, apical segment of LAD, first diagonal, right coronary artery or posterior descending artery, and 0.5 for other segments. In the left dominant coronary system, the multiplying factor of proximal, mid, and distal segment of LCX is 2, 2, and 3.5 respectively, and other segments are consistent with the right dominant coronary system. The final GS is the sum of all the lesion scores. Depending on the median of GS (30.0), the CAD patients were divided into mild CAD group (GS < 30) and severe CAD group (GS≥30).

### Statistical Analysis

Categorical variables were expressed as n (%), normally distributed data and non-normally distributed data of continuous variables were expressed as mean ± standard deviation and median (25th−75th percentile), respectively. The normality of continuous variables was tested by Shapiro-Wilk's test. One-way analysis of variance with *post-hoc* analysis using Student-Newman-Keuls test or Kruskal -Wallis H test with *post-hoc* analysis using Mann-Whitney *U* test was used for the comparison of the continuous variables among the three groups (non-CAD group, mild CAD group, and severe CAD group), independent student's *t*-test or Mann-Whitney *U* test was performed to compare the quantitative variables between two groups (non-severe CAD group and severe CAD group), and Chi-square test was used to analyze categorical variables. Because of the non-normal distribution of variables, Spearman's test was chosen to determine the correlation between two continuous variables. Variables with statistical significance (*P* < 0.05) in univariate logistic regression (Model 1: non-adjusted; Model 2: age and sex-adjusted) were further used in the multivariate logistic regression analysis (Model 3: Multivariate) to clarify the association of serum cytokines and other factors with severe CAD. Then receiver operating characteristic (ROC) curve analysis was performed to assess the IL-12p70, IL-17, HDL-C, and the combination of three biomarkers in predicting severe CAD, and Youden's index was applied to determine the optimal cut-off value. The performance of each independent variable and the models they combined in predicting severe CAD were determined by the area under curve (AUC). All statistical analysis in this research were performed with SPSS software version 26.0 (IBM SPSS, Inc., Chicago, USA), and the results were considered statistically significant if the two-tailed *P* < 0.05.

## Results

### Characteristics of the Study Population

The demographic data and clinical information of the study patients were summarized in Table 1. In the comparison of three groups (non-CAD, mild CAD, and severe CAD), the mild CAD group had a higher proportion of males (*P* = 0.003), smoking (*P* = 0.001), diabetes (*P* = 0.005), and aspirin usage (*P* = 0.037), higher hsCRP level (*P* = 0.001), and lower HDL-C (*P* = 0.019) level than non-CAD group; compared with both non-CAD group and mild CAD group, patients in severe CAD group demonstrated significantly increased in percentage of males (*P* < 0.001 and *P* = 0.046), prevalence of diabetes (*P* < 0.001 and *P* = 0.006) and hsTnI level (*P* < 0.001 and *P* < 0.001), and decreased in HDL-C (*P* < 0.001 and *P* < 0.001) level, besides, severe CAD group showed a higher proportion of smoking (*P* < 0.001) and aspirin usage (*P* < 0.009), higher levels of TG (*P* = 0.004), hsCRP (*P* = 0.004), Scr (*P* < 0.001), and FBG (*P* = 0.001) comparing with the non-CAD group. And in the comparison between non-severe CAD group and severe CAD group, the proportion of males (*P* < 0.001), smoking (*P* = 0.005) and diabetes (*P* < 0.001), and levels of TG (*P* = 0.008), Scr (*P* = 0.001), FBG (*P* = 0.003) and hsTnI (*P* < 0.001) were higher in the severe CAD group, while non-severe CAD group demonstrated higher HDL-C (*P* < 0.001) level.

**Table 1:** Clinical characteristics and serum cytokine levels of patients in each group.

**Group**	**Non-severe CAD**			**Severe CAD**	***P***
	**(GS** **<** **30)**			**(GS≥30, *n* = 245)**	
	**Non-CAD (*n* = 109)**	**Mild CAD (*n* = 148)**	**Total (*n* = 257)**		
Age, years	59.12 ± 9.58	61.05 ± 8.98	60.23 ± 9.27	59.69 ± 11.04	0.229
Male, *n* (%)	55 (50.5)	102 (68.9)^**A**^	157 (61.1)	191 (78.0)^**A**^^**b**^^**C**^	**<0.001**
BMI (kg/m^2^)	25.96 ± 3.59	25.62 ± 3.00	25.76 ± 3.26	26.04 ± 3.47	0.124
Smoking, *n* (%)	23 (21.1)	60 (40.5)^**A**^	83 (32.3)	109 (44.5)^**A**^^**C**^	**<0.001**
Basal diseases, *n* (%)					
Hypertension	65 (59.6)	97 (65.5)	162 (63.0)	153 (62.4)	0.620
Hyperlipidemia	61 (56.0)	93 (62.8)	154 (59.9)	161 (65.7)	0.216
Diabetes	18 (16.5)	47 (31.8)^**A**^	65 (25.3)	112 (45.7)^**A**^^**B**^^**C**^	**<0.001**
Medications, *n* (%)					
Aspirin	49 (45.0)	86 (58.1)^**a**^	135 (52.5)	147 (60.0)^**A**^	**0.027**
Statin	60 (55.5)	92 (62.2)	152 (59.1)	160 (65.3)	0.185
ACEI/ARB	29 (26.6)	42 (28.4)	71 (27.6)	74 (30.2)	0.778
Biochemical parameters					
TG (mmol/L)	1.31 (0.95–1.75)	1.36 (1.08–1.98)	1.35 (1.00–1.82)	1.46 (1.14–2.10)^**A**^^**C**^	**0.011**
TC (mmol/L)	3.71 (3.27–4.80)	3.78 (3.22–4.46)	3.74 (3.25–4.54)	3.66 (3.14–4.23)	0.238
HDL-C (mmol/L)	1.07 (0.94–1.22)	1.00 (0.87–1.17)^**a**^	1.03 (0.90–1.18)	0.88 (0.78–1.05)^**A**^^**B**^^**C**^	**<0.001**
LDL-C (mmol/L)	2.00 (1.67–2.87)	2.11 (1.59–2.68)	2.07 (1.62–2.76)	2.00 (1.61–2.46)	0.182
nonHDL (mmol/L)	2.64 (2.22–3.62)	2.75 (2.18–3.44)	2.67 (2.19–3.50)	2.67 (2.18–3.25)	0.655
hsCRP (mg/l)	0.79 (0.46–1.65)	1.18 (0.65–3.06)^**A**^	1.03 (0.55–2.35)	1.12 (0.63–2.57)^**A**^	**0.003**
Hcy (μmol/l)	13.10 (11.45–15.25)	13.70 (12.10–15.10)	13.30 (11.70–15.15)	13.90 (11.80–16.00)	0.168
Urea (mmol/L)	5.16 (4.15–6.25)	5.21 (4.49–6.27)	5.20 (4.39–6.25)	5.31 (4.57–6.51)	0.255
Scr (μmol/L)	66.6 (57.3–76.8)	70.1 (62.5–79.3)	69.1 (59.7–77.9)	72.7 (64.0–84.0)^**A**^^**C**^	**0.001**
eGFR (ml/min)	97.4 (89.8–102.8)	94.9 (85.0–102.7)	96.2 (87.1–102.8)	95.4 (85.0–104.4)	0.399
UA (μmol/L)	326.7 (273.7–386.9)	329.1 (277.0–387.2)	328.1 (275.2–387.0)	342.6 (277.7–401.6)	0.727
FBG (mmol/L)	5.04 (4.66–5.78)	5.29 (4.77–6.31)	5.14 (4.75–6.17)	5.63 (4.81–7.03)^**A**^^**C**^	**0.002**
hsTnI (pg/ml)	2.70 (1.25–5.00)	2.90 (1.50–4.90)	2.90 (1.40–4.95)	5.00 (2.10–12.00)^**A**^^**B**^^**C**^	**<0.001**
Cytokines (pg/ml)					
IL-1β	1.62 (0.68–3.02)	2.11 (0.62–3.34)	1.91 (0.66–3.13)	1.69 (0.57–2.96)	0.294
IL-2	0.75 (0.44–1.23)	0.80 (0.42–1.30)	0.76 (0.42–1.27)	0.66 (0.41–1.09)	0.246
IL-4	1.38 (0.87–1.95)	1.32 (0.70–1.85)	1.36 (0.77–1.88)	1.12 (0.59–1.64)^**A**^^**b**^^**C**^	**0.007**
IL-5	1.03 (0.47–1.82)	0.99 (0.45–1.63)	1.00 (0.45–1.69)	0.97 (0.41–1.63)	0.885
IL-6	3.83 (2.37–6.43)	4.38 (2.29–7.05)	4.01 (2.29–6.87)	4.31 (2.73–7.44)	0.257
IL-8	41.50 (21.18–96.05)	51.70 (22.47–113.73)	46.34 (21.84–108.21)	47.83 (24.09–119.06)	0.578
IL-10	2.09 (1.58–3.04)	2.13 (1.46–3.41)	2.12 (1.52–3.15)	2.12 (1.43–2.91)	0.534
IL-12p70	1.92 (1.28–2.54)	2.01 (1.40–2.69)	1.96 (1.33–2.59)	1.40 (0.83–2.09)^**A**^^**B**^^**C**^	**<0.001**
IL-17	4.22 (2.50–7.81)	4.19 (1.97–9.06)	4.22 (2.08–8.34)	2.50 (0.67–4.86)^**A**^^**B**^^**C**^	**<0.001**
TNF-α	1.83 (0.92–2.97)	1.73 (1.02–3.46)	1.78 (0.94–3.14)	1.76 (0.91–2.72)	0.491
IFN-α	1.70 (0.96–2.48)	1.60 (0.83–2.69)	1.67 (0.88–2.59)	1.50 (0.71–2.18)^**a**^^**c**^	**0.037**
IFN-γ	1.27 (0.70–1.99)	1.11 (0.48–1.92)	1.23 (0.56–1.95)	1.14 (0.54–1.89)	0.594
Gensini score	2.5 (0–5)	18.5 (12–23.5)^**A**^	10 (3–20)	60 (44–87)^**A**^^**B**^^**C**^	**<0.001**

*Values are expressed as percentages, mean ± SD, or median (25th−75th percentile). Bold values signifies the statistical significance in the comparison of three groups (non-CAD, mild CAD, and severe CAD).*

a *<0.05 vs. Non-CAD group;*

b *p <0.05 vs. Mild CAD group;*

c *p <0.05 vs. Total Non-severe CAD group.*

A *p <0.01 vs. Non-CAD group;*

B *p <0.01 vs. Mild CAD group;*

C *p <0.01 vs. Total Non-severe CAD group*.

### Serum Cytokine Levels Under Different Groupings

**Figure 1:**
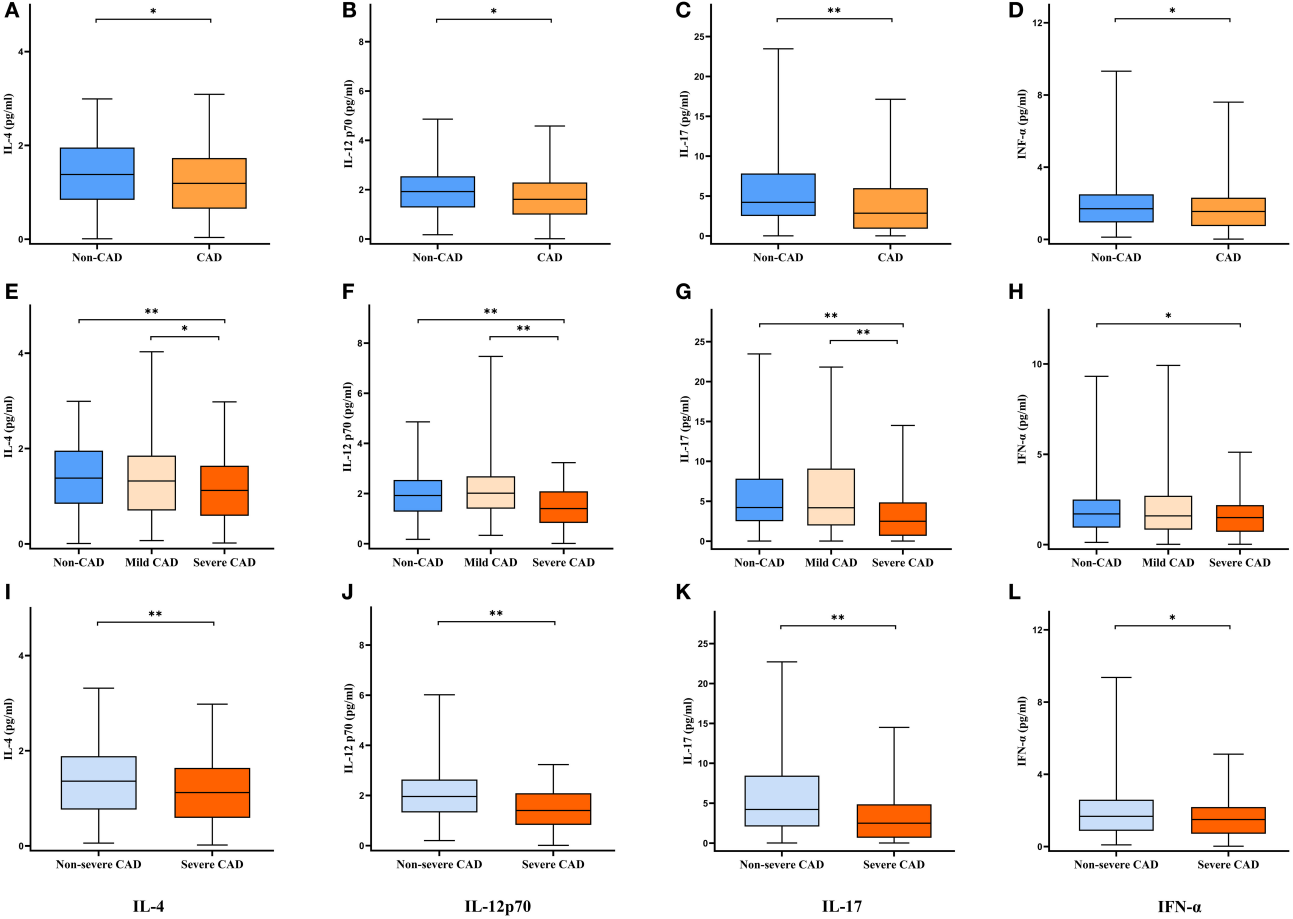
Serum cytokine levels under different groupings. Data are expressed as median with 25th and 75th percentiles. **(A–D)** Non-CAD group vs. CAD group. **(E–H)** Non-CAD group vs. Mild CAD group vs. Severe CAD group. **(I–L)** Non-severe CAD group vs. Severe CAD group. *P < 0.05, **P < 0.01.

The results of all serum cytokine assay were shown in Table 1, there were no statistical differences in serum cytokine levels among the three groups except for IL-4, IL-12p70, IL-17, and IFN-α, and the levels of IL-4, IL-12p70, IL-17, and IFN-α under different groupings were demonstrated in Figure 1. Compared with the non-CAD group, patients with CAD had lower IL-4 (*P* = 0.040), IL-12p70 (*P* = 0.010), IL-17 (*P* < 0.001), and IFN-α (*P* = 0.048) levels. In the comparison among the non-CAD group, mild CAD group, and severe CAD group, the expression levels of IL-4 (*P* = 0.005 and *P* = 0.017), IL-12p70 (*P* < 0.001 and *P* < 0.001), and IL-17 (*P* < 0.001 and *P* < 0.001) were significantly lower in severe CAD group than both in non-CAD group and mild CAD group, IFN-α level are lower in severe CAD group than in non-CAD group (*P* = 0.014) but no statistical difference between severe CAD group and mild CAD group (*P* = 0.109), and all the cytokine levels did not differ significantly between non-CAD group and mild CAD group. IL-4 (*P* = 0.002), IL-12p70 (*P* < 0.001), IL-17 (*P* < 0.001), and IFN-α (*P* = 0.015) levels were significantly reduced in the severe CAD group comparing with the non-severe CAD group.

### Correlation Between Serum Cytokine and CAD Severity

**Figure 2:**
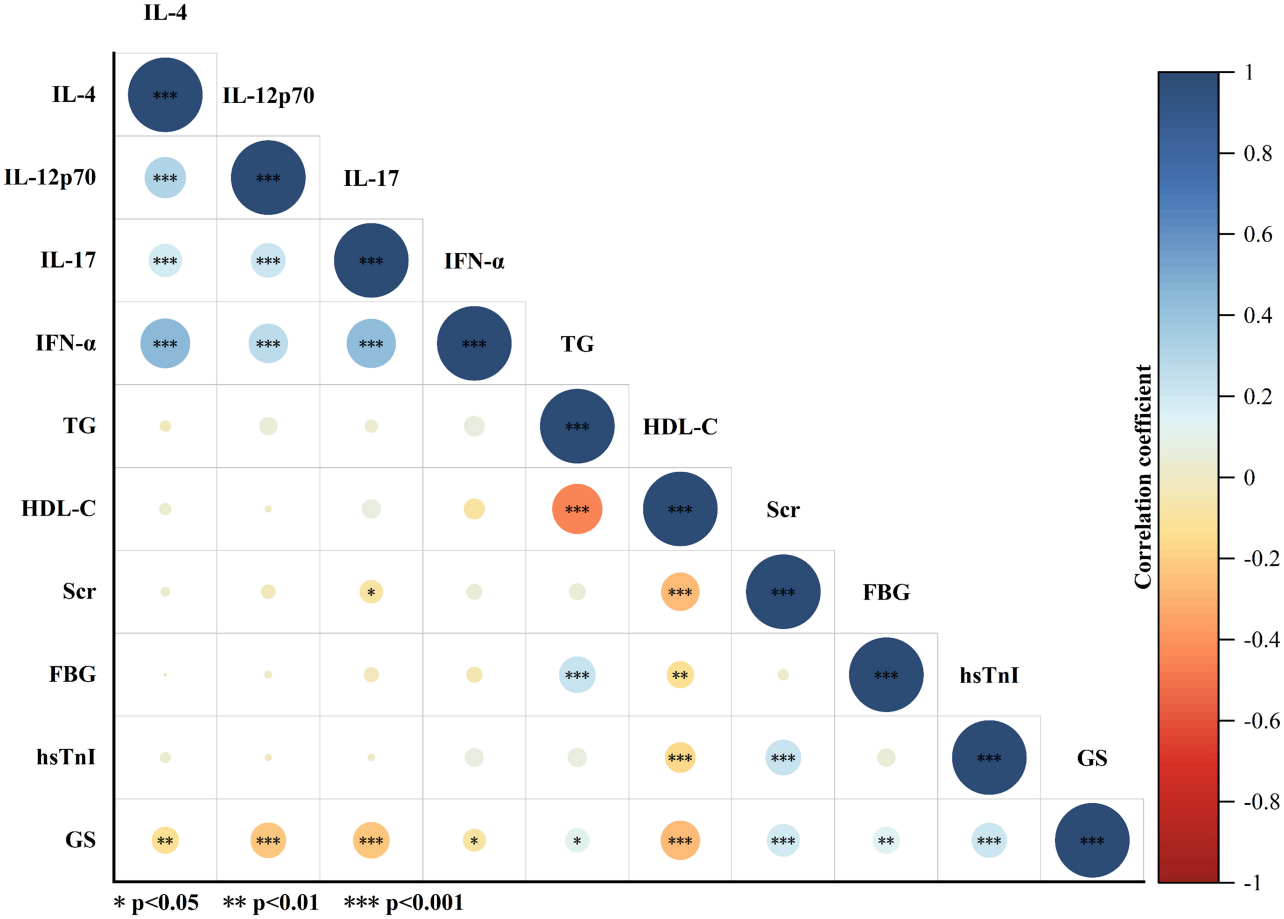
Pearson correlation analysis of variables with statistically significant difference between non-severe CAD group and severe CAD group. The area and color of the circle represent the value of the correlation coefficient. The larger the area of the circle, the larger the absolute value of the correlation coefficient; the color of the circle corresponds to the value of the correlation coefficient on the color scale. *P < 0.05, **P < 0.01, ***P < 0.001.

**Figure 3:**
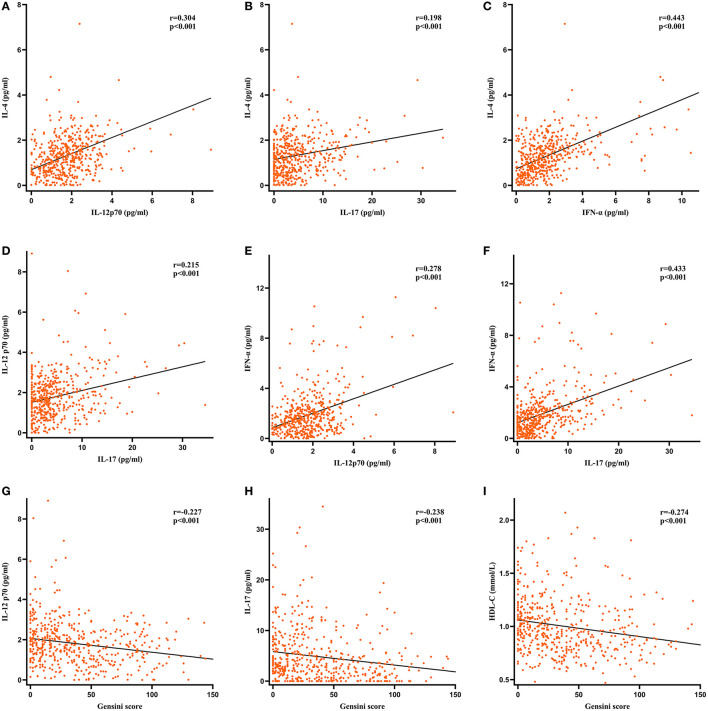
Scatterplots of serum cytokines levels, HDL-C and Gensini score. r, correlation coefficient. **(A–F)** Correlation among IL-4, IL-12p70, IL-17 and IFN-α. **(G–I)** The levels of IL-12p70, IL-17 and HDL-C were negatively correlated with Gensini score.

As shown in Figure 2, Spearman test was performed to evaluate the correlation among GS, serum cytokine levels, and other parameters with statistically significant difference between the non-severe CAD group and the severe CAD group in Table 1. The results of Spearman test revealed that IL-4 level was positively correlated with the levels of IL-12p70 (r = 0.304, *P* < 0.001), IL-17 (r = 0.198, *P* < 0.001), and IFN-α (r = 0.443, *P* < 0.001), the levels of IL-17 (r = 0.215, *P* < 0.001) and IFN-α (r = 0.278, *P* < 0.001) were weakly associated with IL-12p70 level, and there was a positive correlation between serum IFN-α level and IL-17 level (r = 0.433, *P* < 0.001). The levels of IL-12p70 (r = −0.227, *P* < 0.001), IL-17 (r = −0.238, *P* < 0.001) and HDL-C (r = −0.274, *P* < 0.001) had a weakly negative correlation with GS. The results mentioned above were further demonstrated in Figure 3. Other parameters like IFN-α (r = −0.093, *P* = 0.038), TG (r = 0.107, *P* = 0.017), Scr (r = 0.193, *P* < 0.001), FBG(r = 0.130, *P* < 0.004) and hsTnI (r = 0.219, *P* < 0.001) are also statistically correlated with GS but had a low correlation coefficient.

### Multivariate Logistic Regression Analyses of Severe CAD

To explore independent predictors for severe CAD, firstly, we analyzed the relationship of various parameters and severe CAD by univariate logistic regression. As indicated in Table 2, gender (OR = 2.253, 95%CI: 1.521–3.336, *P* < 0.001), smoking (OR = 1.680, 95%CI: 1.169–2.416, *P* = 0.005), diabetes (OR = 2.487, 95%CI: 1.706–3.627, *P* < 0.001), TG (OR = 1.323, 95%CI: 1.101–1.590, *P* = 0.003), HDL-C (OR = 0.184, 95%CI: 0.082–0.412, *P* < 0.001), FBG (OR = 1.146, 95%CI: 1.056–1.243, *P* = 0.001), IL-4 (OR = 0.750, 95%CI: 0.607–0.927, *P* = 0.007), IL-12p70 (OR = 0.551, 95%CI: 0.452–0.672, *P* < 0.001), IL-17 (OR = 0.907, 95%CI: 0.870–0.946, *P* < 0.001) and IFN-α (OR = 0.840, 95%CI: 0.750–0.942, *P* = 0.003) demonstrated statistically significant correlated with severe CAD. After adjusted for age and gender, diabetes (OR = 2.550, 95%CI: 1.733–3.751, *P* < 0.001), TG (OR = 1.340, 95%CI: 1.109–1.620, *P* = 0.002), HDL-C (OR = 0.214, 95%CI: 0.096–0.474, *P* < 0.001), FBG (OR = 1.152, 95%CI: 1.061–1.251, *P* = 0.0013), IL-4 (OR = 0.747, 95%CI: 0.605–0.923, *P* = 0.007), IL-12p70 (OR = 0.566, 95%CI: 0.464–0.691, *P* < 0.001), IL-17 (OR = 0.913, 95%CI: 0.876–0.952, *P* < 0.001) and IFN-α (OR = 0.837, 95%CI: 0.746–0.939, *P* = 0.002) were still correlated with severe CAD. Subsequent multivariate logistic regression (gender, diabetes, TG, HDL-C, FBG, IL-4, IL-12p70, IL-17 and IFN-α were taken into account) revealed that IL-12p70 (OR = 0.572, 95%CI: 0.454–0.720, *P* < 0.001), IL-17 (OR = 0.930, 95%CI: 0.886–0.976, *P* = 0.003) and HDL-C (OR = 0.232, 95%CI: 0.091–0.593, *P* = 0.002) were independent predictors for severe CAD. In addition, we also confirmed the independence of two classic risk factors: gender (OR = 1.658, 95%CI: 1.048–2.624, *P* = 0.031) and diabetes (OR = 2.444, 95%CI: 1.509–3.958, *P* < 0.001) by multivariate logistic regression.

**Table 2:** Logistic regression analysis to predict severe CAD.

**Variables**	**Model 1: No adjustment**		**Model 2: Age and sex-adjusted**		**Model 3: Multivariate**	
	**OR (95%CI)**	***P***	**OR (95%CI)**	***P***	**OR (95%CI)**	***P***
Age	0.995 (0.978–1.012)	0.546	-	-	-	-
Male vs. Female	2.253 (1.521–3.336)	**<0.001**	-	-	1.658 (1.048–2.624)	**0.031**
Smoking	1.680 (1.169–2.416)	**0.005**	1.204 (0.791–1.833)	0.385	-	-
Diabetes	2.487 (1.706–3.627)	**<0.001**	2.550 (1.733–3.751)	**<0.001**	2.444 (1.509–3.958)	**<0.001**
TG	1.323 (1.101–1.590)	**0.003**	1.340 (1.109–1.620)	**0.002**	1.182 (0.947–1.475)	0.139
HDL-C	0.149 (0.069–0.318)	**<0.001**	0.184 (0.082–0.412)	**<0.001**	0.232 (0.091–0.593)	**0.002**
Scr	1.009 (1.000–1.019)	0.057	1.004 (0.995–1.012)	0.391	-	-
FBG	1.126 (1.040–1.219)	**0.004**	1.146 (1.056–1.243)	**0.001**	1.002 (0.909–1.104)	0.971
hsTnI	1.004 (1.000–1.008)	0.071	1.000(1.000–1.001)	0.210	-	-
hsCRP	0.987 (0.932–1.046)	0.667	0.984 (0.928–1.044)	0.597	-	-
IL-4	0.750 (0.607–0.927)	**0.008**	0.747 (0.605–0.923)	**0.007**	0.971 (0.750–1.256)	0.820
IL-12p70	0.551 (0.452–0.672)	**<0.001**	0.566 (0.464–0.691)	**<0.001**	0.572 (0.454–0.720)	**<0.001**
IL-17	0.907 (0.870–0.946)	**<0.001**	0.913 (0.876–0.952)	**<0.001**	0.930 (0.886–0.976)	**0.003**
IFN-α	0.840 (0.750–0.942)	**0.003**	0.837 (0.746–0.939)	**0.002**	0.977 (0.848–1.124)	0.741

*The dependent variable of logistics regression is whether patients have severe CAD, and the independent variables are age, sex, BMI, smoking, diabetes, TG, HDL-C, Scr, FBG, hsTnI, hsCRP, IL-4, IL-12p70, IL-17, IFN-α.*

*Model 1: Univariate logistic regression.*

*Model 2: Logistic regression after adjusted age and gender.*

*Model 3: Parameters with p <0.05 in Model 2 were subsequently entered into multivariate regression analysis.*

*Bold values signifies the statistical significance in logistics regression*.

### ROC Curve Analysis of IL-12p70, IL-17, and HDL-C in Predicting Severe CAD

**Figure 4:**
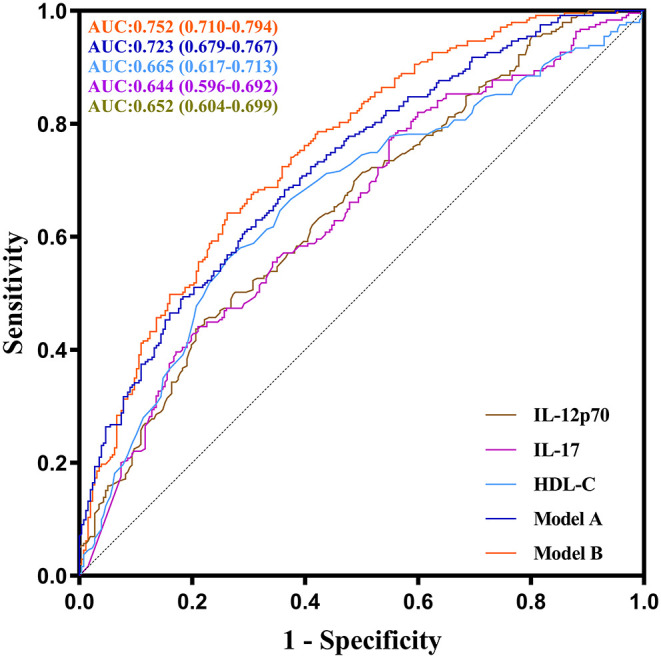
ROC curves of IL-12p70, IL-17, HDL-C and their combination showing different abilities to predict severe CAD. The AUC of the serum IL-12p70 level was 0.652 (95 %CI: 0.604–0.699, *P* < 0.001), the AUC of the serum IL-17 level was 0.644 (95 %CI: 0.596–0.692, *P* < 0.001), the AUC of the HDL-C level was 0.665 (95 %CI: 0.617–0.713, *P* < 0.001), the AUC of the model A(combined IL-12p70, IL-17 and HDL-C) was 0.723 (95 %CI: 0.679–0.767, *P* < 0.001), and the AUC of the model B (combined IL-12p70, IL-17, HDL-C, gender and diabetes) was 0.752 (95 %CI: 0.710–0.794, *P* < 0.001).

ROC curve analysis was performed to evaluate the performance of IL-12p70, IL-17, and HDL-C in predicting severe CAD. As shown in Figure 4, the AUC of ROC curve for IL-12p70, IL-17 and HDL-C predicting severe CAD alone were 0.652 (95%CI:0.604–0.699, *P* < 0.001), 0.644 (95%CI:0.596–0.692, *P* < 0.001) and 0.665 (95%CI: 0.617–0.713, *P* < 0.001), respectively. And the AUC can increase to 0.723 (95%CI: 0.710–0.794, *P* < 0.001) by combining the three independent factors (Model A). Furthermore, the multivariate model (Model B) combining sex, diabetes, IL-12p70, IL-17, and HDL-C showed further improved ability in predicting severe CAD (AUC = 0.752, 95%CI: 0.710–0.794, *P* < 0.001).

### Predictive Performance of IL-12p70, IL-17, and HDL-C for Severe CAD

**Figure 5:**
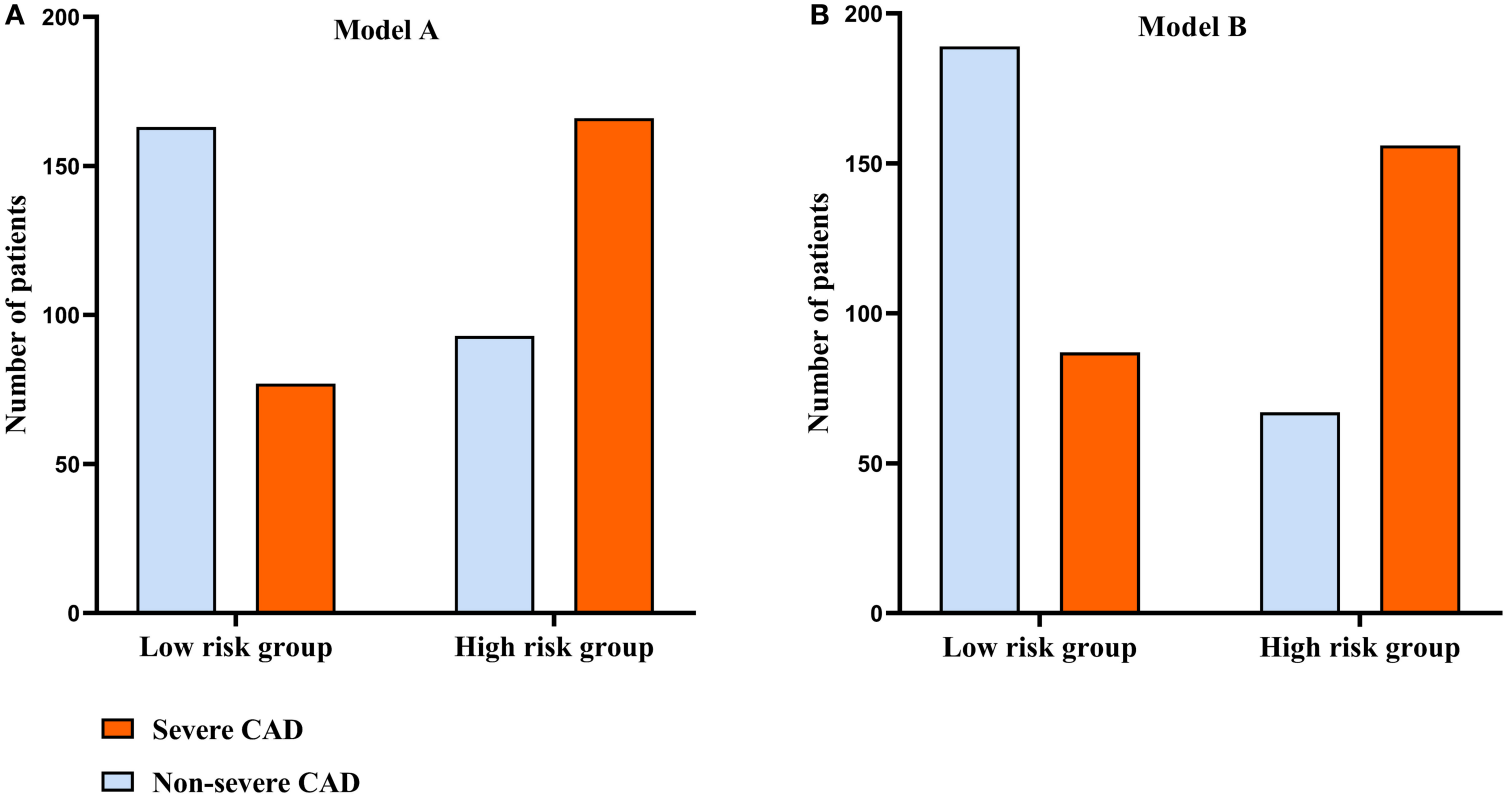
Comparison of the distribution of patients between the groups classified according to the optimal cut-off value of the model A and model B. **(A)** The distribution of patients classified by model A. **(B)** The distribution of patients classified by model B.

In Figure 5, to assess the predictive performance of Model A and Model B, the optimal cut-off value of Model A and Model B was used to divide all patients into two groups (high risk group and low risk group), and the high risk group had much more patients with severe CAD comparing with the low risk group. To further elucidate the real predictive abilities of our factors, in Table 3, we calculated the sensitivity, specificity, positive predictive values, negative predictive values, false-positive values, false-negative values, likelihood ratio for positive finding, and likelihood ratio for negative finding analysis of IL-12p70 (cut-off value: 1.265 pg/ml), IL-17 (cut-off value: 1.950 pg/ml), HDL-C (cut-off value:0.905 mmol/L), Model A and Model B. Compared with IL-12p70 and IL-17, HDL-C showed lower specificity (73.8 vs. 77.8% and 79.0%) but higher sensitivity (56.0 vs. 45.3% and 44.1%). Model A had the highest sensitivity (68.3%), but lower specificity (63.7%). Model B had a higher sensitivity than IL-12p70 and IL-17, HDL-C (64.2 vs. 45.3%, 44.1 and 56.0%), and a higher specificity than Model A (73.8 vs. 63.7%). Moreover, Model B demonstrated highest positive predictive value (70.0%), negative predictive value (68.5%), and positive likelihood ratios (2.452), and lowest negative likelihood ratios (0.484).

**Table 3:** Diagnosis accuracy assessment of IL-12p70, IL-17, HDL-C and their combination in predicting severe CAD.

	**IL-12p70**	**IL-17**	**HDL-C**	**Model A**	**Model B**
Sensitivity (%)	45.3 (39.0–51.8)	44.1 (37.8–50.5)	56.0 (49.5–62.3)	68.3(62.0–74.0)	64.2 (57.8–70.2)
Specificity (%)	77.8 (72.1–82.6)	79.0 (73.4–83.7)	73.8 (67.9–79.0)	63.7 (57.4–69.5)	73.8 (67.9–79.0)
Positive predictive value (%)	66.1 (58.3–73.1)	66.7 (58.8–73.8)	67.0 (60.0–73.3)	64.1 (57.9–69.9)	70.0 (63.4–75.8)
Negative predictive value (%)	59.9 (54.4–65.1)	59.7 (54.3–64.9)	63.8 (58.1–69.3)	67.9 (61.6–73.7)	68.5 (62.6–73.8)
False positive (%)	33.9 (26.9–41.7)	33.4 (26.2–41.2)	33.0 (26.7–40.0)	35.9 (30.1–42.1)	30.0 (24.2–36.6)
False negative (%)	40.1 (34.9–45.6)	40.3 (35.1–45.7)	36.1 (30.7–41.9)	32.1 (26.3–38.4)	31.5 (26.2–37.4)
Positive likelihood ratios	2.043 (1.564–2.668)	2.098 (1.592–2.764)	2.138 (1.692–2.702)	1.880 (1.565–2.259)	2.452 (1.956–3.075)
Negative likelihood ratios	0.703 (0.626–0.789)	0.708 (0.632–0.793)	0.596 (0.516–0.689)	0.498 (0.412–0.601)	0.484 (0.409–0.575)

*Model A: the combination of IL-12p70, IL-17, and HDL-C.*

*Model B: the combination of combined IL-12p70, IL-17, HDL-C, gender, and diabetes*.

## Discussion

CAD remains the leading cause of all global deaths (20). The severity and clinical outcome of CAD were closely linked, all-cause mortality increased with the increasing severity of CAD, and patients with more severe CAD had a higher risk of cardiovascular death and myocardial infarction (2). However, a significant number of patients with CAD only exhibited atypical or absent symptoms of chest discomfort even though the coronary lesions were serious, patients often did not pay enough attention to it, and they are also easily overlooked in clinical work (21, 22). Therefore, it is meaningful to identify patients with high severity of CAD from people suspected to have CAD by identifying the predictors of severe CAD, and would greatly improve the prevention, diagnosis, and treatment of CAD. In this study, we revealed a close association between serum cytokines and the severe CAD determined by GS. Patients in the severe CAD group had lower levels of IL-4, IL-12p70, IL-17, and IFN-α than the non-severe CAD group. And in the multivariate logistic regression analyses of severe CAD, the lower levels of IL-12p70, IL-17, and HDL-C were significantly associated with severe CAD after adjusting for other related factors. A novel predictive model for severe CAD was constructed by combining IL-12p70, IL-17, HDL-C, and other cardiovascular clinical risk factors, and this model demonstrated good performance on identifying severe CAD patients with high GS from patients suspected to have CAD in the ROC analysis, with a sensitivity and specificity of 64.2 and 73.8%, respectively.

We excluded patients with AMI from our study population for the following two reasons. On the one hand, most AMI can be quickly and accurately identified by the more obvious symptoms of chest pain, electrocardiograms, and markers of myocardial injury, even in primary health facilities with limited medical resources, and then, percutaneous coronary intervention or thrombolytic therapy was chosen depending on the local medical conditions and the time of AMI occurred. Myocardial injury markers such as troponin had high sensitivity and specificity in reflecting cardiac injury and predicting myocardial infarction, which was unmatched by serum cytokines. Therefore, the conclusions drawn from the study including patients with AMI could not improve clinical practices. On the other hand, when patients suffered AMI, a large number of cytokines would be released into the blood, which could cause dramatic changes in the levels of various cytokines within a short period of time. Many previous studies exploring the association between serum cytokines and CAD had shown that cytokine levels of patients in the AMI group were significantly different from other subgroups of CAD (18, 23). The changes in serum cytokines levels after AMI reflected acute plaque rupture and myocardial injury more rather than the chronic progression of atherosclerotic plaques. Therefore, the inclusion of patients with AMI in the study using the levels of cytokines to predict the severity of CAD might make the results inaccurate.

We used CAG which was the gold standard for CAD diagnosis to clarify the presence of disease and further assessed the severity of coronary lesions by GS. The GS provided an accurate assessment for the severity of coronary atherosclerotic plaque burden, and thus could predict the risk of future cardiovascular events (24). By CAG and GS, we classified patients suspected to have CAD into different groups. In our study, the serum levels of IL-4, IL-12p70, IL-17, and IFN-α in the severe CAD group were significantly lower than the non-severe CAD group, while their levels were not statistically different between non-CAD group and mild CAD group. The roles of IL-4 and IL-17 in CAD were controversial in previous studies. It is reported that IL-4 played a protective role in atherosclerosis (25), but some studies demonstrated that IL-4 levels were higher in patients with CAD than normal people (26, 27). The same is true for IL-17, IL-17 was found to promote the development of atherosclerotic plaques by upregulating a variety of pro-inflammatory mediators and cytokines (28), and the circulating level of IL-17 were positively correlated with GS in patients with CAD (29), besides, the relationship found between the amount of visceral fat and circulating levels of IL-17-related chemokine eotaxin on the one hand, and intima-media thickness which is the marker of early atherosclerosis on the other, might suggest that IL-17 play an indirect role in promoting early subclinical atherosclerosis in obese patients (30). However, it has also been suggested that IL-17 played an athero-protective effect by reducing the formation of atherosclerotic plaques (31), and a lower level of IL-17 was associated with a higher risk of cardiovascular events (32). Our study data showed that the levels of IL-4 and IL-17 levels decreased in the severe CAD group comparing to the non-CAD group and mild CAD group, this result preferred that IL-4 and IL-17 might have a protective effect on the development of atherosclerosis and it was also possible that they played different roles at different stages of atherosclerosis, but at least the low levels of circulating IL-4 and IL-17 in patients suspected to have CAD were suggestive of more severe coronary lesions. IL-12p70 was the bioactive IL-12, and it was a dimeric protein and consisted of two disulfide-linked subunits: p35 and p40 (33). Previous studies have generally agreed that IL-12 promoted the formation and development of atherosclerosis, and clinical data showed that patients with CAD exhibit higher serum concentration of IL-12 (34, 35). However, in the current study, patients with severe CAD demonstrated lower serum level of IL-12p70, this might be due to the fact that previous studies did not exclude patients with AMI, and serum levels of IL-12 were substantially increased in patients suffering from AMI when compared to patients with unstable angina pectoris or healthy controls (36), but the increase in IL-12 level at this time reflected acute myocardial injury rather than the chronic progression of atherosclerotic plaques. Therefore, when we excluded patients with AMI and had a relatively large sample size, we arrived at the opposite conclusion that patients with a reduced IL-12p70 level might have severe CAD. In fact, there had been a few studies that had come to different conclusions, an observational study had shown that there was no association between IL-12 level and cardiovascular event, including AMI, unstable angina pectoris, and ischemic stroke (37), and it had been found that IL-12 could play an anti-inflammatory role in some inflammatory microenvironment (38, 39). IFN-α was thought to have a potential atherogenic effect in SLE patients (10), but the role of IFN-α in atherosclerosis had rarely been reported in patients without SLE. And Our study found that patients with severe CAD had a lower serum concentration of IFN-α. Spearman correlation analyses demonstrated that there were different degrees of positive correlations among IL-4, IL-12p70, IL-17, and IFN-α, and it suggested that they might have synergistic effects in CAD. In addition, other clinical factors, such as gender, smoking, diabetes, TG, HDL-C, Scr, FBG, and hsTnI also showed statistical differences between the severe CAD group and the non-severe CAD group in this study, which were consistent with previous studies. The association between these variables and GS was explored by Spearman correlation analyses, and circulating levels of IL-12p70, IL-17, and HDL-C were found to be weakly correlated with the GS. The reduced level of HDL-C was recognized as a strong independent predictor of CAD, Framingham Heart Study had revealed a strong inverse correlation between HDL-C levels and the risk of CAD, even with a normal LDL-C level (40). And the anti-atherogenic function of HDL-C was mainly achieved by mediating reverse cholesterol transport (40).

Univariate and multivariate logistic regression analysis were performed to confirm the value of serum cytokines and other factors in predicting severe CAD. Finally, IL-12p70, IL-17, HDL-C, and two classical risk factors (gender and diabetes) were determined as the independent predictors of severe CAD after adjustment for age, gender, and other associated factors. To further evaluate their performance in predicting severe CAD from patients with suspected CAD, ROC curve analysis was performed. The ideal predictors should be both sensitive and specific for the disease diagnosis, but in practice using only a single biomarker to predict a complex disease usually had an unsatisfactory performance, with poor sensitivity (41). When one of IL-12p70, IL-17, and HDL-C was used alone to predict severe CAD, the results obtained showed high specificity but low sensitivity. To improve the predicting performance, we further construct model A and model B by combining IL-12p70, IL-17, and HDL-C, and the sensitivity rose to 68.3 and 64.2%, respectively, at the same time, model B retains a good specificity of 73.8%.

The current study provided a predictive model to identify severe CAD patients with a high GS from those with suspected CAD but not AMI. Although it was not as accurate as CAG which was the gold standard for CAD diagnosis, our models had the advantage of low cost, low-risk, convenient, and requiring less in medical equipment and professional skills of medical staff. And it might be a practical tool to improve the diagnosis and treatment of CAD, especially for primary health care facilities with limited cardiac catheterization resources. In determining the predictors of CAD severity, we performed the analysis in the entire population of suspected CAD, rather than only in patients with CAD as previous studies had done, which was more in line with clinical reality. Most patients with severe CAD could be identified in health care facilities without cardiac catheterization room by this model, and these patients could then be strongly advised to go to a hospital with relevant medical equipment for CAG and subsequent treatment. For those without severe CAD, other non-invasive examinations or further follow-ups might also be chosen in addition to CAG, and it would reduce the financial burden on patients and the additional risks associated with the invasive examination.

The limitations of our study should also be considered. Firstly, the study patients enrolled in a single center only, and most of them came from the northern provinces of China, joint study of multiple centers in different geographic areas might be more convincing. Secondly, previous studies had shown that IL-1β, IL-6, and TNF-α are associated with ACS, but no association between these cytokines and the severity of CAD was found in the present study, and it was probably due to the exclusion of AMI patients and the lack of a large sample size, although the present study had a relatively large number of subjects, it was still inadequate compared to the large coronary heart disease population, the larger sample size was needed to make the findings generalizable. Thirdly, the percentage of male gender and diabetes presence in the three groups is completely different, this may lead to biased data, although gender and diabetes are important risk factors for coronary heart disease. Fourth, besides the 12 cytokines tested here, there were other cytokines involved in the formation and progression of atherosclerosis and they might also correlate with the severity of CAD. Finally, we only conducted a cross-sectional study and did not follow patients over time to record follow-up information, and further prospective studies are needed to validate the role of cytokines in guiding the diagnosis and treatment of CAD.

## Data Availability Statement

The datasets presented in this article are not readily available because of the protection of personal data and privacy restrictions. Requests to access the datasets should be directed to MZ, zhangming2279@hotmail.com.

## Ethics Statement

The studies involving human participants were reviewed and approved by the Medical Ethics Committee of Beijing Anzhen Hospital. The patients/participants provided their written informed consent to participate in this study.

## Author Contributions

SL, CW, YY, and MZ conceived and designed the study. CW, JG, MH, and YW collected clinical data from patients. SL, CW, JG, LL, and YW analyzed the data. YQ and MZ provided significant input to the manuscript. SL, CW, JG, and YY wrote the manuscript with input from all authors. All authors contributed to the article and agree to be accountable for the content of the work.

## Funding

This work was supported by the National Natural Science Foundation of China (No. 82170453).

## Conflict of Interest

The authors declare that the research was conducted in the absence of any commercial or financial relationships that could be construed as a potential conflict of interest.

## Publisher's Note

All claims expressed in this article are solely those of the authors and do not necessarily represent those of their affiliated organizations, or those of the publisher, the editors and the reviewers. Any product that may be evaluated in this article, or claim that may be made by its manufacturer, is not guaranteed or endorsed by the publisher.
